# Levels and Rates of Physical Activity in Older Adults with Multiple Sclerosis

**DOI:** 10.14336/AD.2015.1025

**Published:** 2016-05-27

**Authors:** Rachel E. Klaren, Emerson Sebastiao, Chung-Yi Chiu, Dominique Kinnett-Hopkins, Edward McAuley, Robert W. Motl

**Affiliations:** Department of Kinesiology & Community Health, University of Illinois at Urbana-Champaign, USA.

**Keywords:** accelerometry, physical activity, older adults, multiple sclerosis

## Abstract

There is much evidence supporting the safety and benefits of physical activity in adults with multiple sclerosis (MS) and recent evidence of beneficial effects on physical function in older adults with MS. However, there is very little known about physical activity participation in older adults with conditions such as MS. This study compared levels of physical activity (i.e., sedentary behavior, light physical activity (LPA), and moderate-to-vigorous physical activity (MVPA)) and rates of meeting public health guidelines for MVPA (i.e., ≥30 min/day) among young (i.e., ages 20-39 years), middle-aged (i.e., ages 40-59 years) and older adults (i.e., ages ≥60 years) with MS. The sample included 963 persons with MS who provided demographic and clinical information and wore an accelerometer for a 7-day period. The primary analysis involved a between-subjects ANOVA on accelerometer variables (i.e., accelerometer wear time; number of valid days; sedentary behavior in min/day; LPA in min/day; and MVPA in min/day). Collectively, our data indicated that older adults with MS engaged in less MVPA and more sedentary behavior than middle-aged and young adults with MS. Such results highlight the importance of developing physical activity interventions as an effective means for managing the progression and consequences of MS in older adults.

Of the estimated 400,000 adults living with multiple sclerosis (MS) in the United States, approximately 32% are between the ages of 55-64 years and 14% are 65 years of age or older [1]. There is additional evidence of a shift in the peak prevalence of MS among older age groups (e.g., in Manitoba, the peak prevalence was at 35-39 years of age, with no documented cases beyond an age of 64 years, in 1984; by 2004, peak prevalence was at 55-59 years of age, with cases of MS documented beyond 80 years of age) [2]. This represents a “greying” of the MS population that coincides with both increased survival of those with MS and the shifting demographic landscape worldwide. That is, there are greater numbers of older adults living with MS than ever before.

This growing cohort of older adults with MS undergoes normal age-related declines in physical and psychological functioning that are seemingly compounded by the disease and its progression [3,4]. Older adults with MS often report poor health status and functioning, suicide ideation, depression, loneliness, cognitive difficulty, and dependence for activities of daily living [5-10]. There is additional evidence of a faster rate of disability progression among older than younger adults with MS [11], and older age is a primary predictor of reaching disability milestones in MS (e.g., median age for unilateral assistance during walking is nearly 65 years) [12].

Little is known about predicting and managing the progression and consequences of MS in older adults. For example, there are 13 FDA-approved disease-modifying agents that represent the first line of therapy primarily for younger and middle-aged adults with relapsing-remitting MS (RRMS); these agents have not been systematically examined in older adults with MS [3,4,13]. Some data suggest that disease-modifying agents might have no association with disability progression in older adults with MS [14]. This underscores the need for new approaches targeting healthy lifestyle behaviors, such as physical activity, in older adults with MS [15].

There is much evidence supporting the safety and benefits of physical activity in adults with MS [16,17] and recent evidence of beneficial effects on physical function in older adults with MS [18]. Physical activity participation may further confer a potential restorative effect on functional limitations and disability as well as a protective effect on the rate of disability progression in older adults with MS [19-21]. To date, there is very little known about physical activity participation and its possible benefits in older adults with conditions such as MS [22].

Overall, only a small proportion of persons with MS engage in levels of physical activity recommended for public health benefits. For example, one study objectively quantified levels and rates of physical activity in persons with MS and demonstrated persons with MS engaged in approximately 13 minutes less moderate-to-vigorous physical activity (MVPA) per day than controls, and only 20% of persons with MS meet public health guidelines for MVPA (i.e., ≥30 min/day) [23]. Another study identified persons with MS were 2.5 times more likely to report insufficient physical activity and 2.3 times less likely to report sufficient physical activity compared with controls [24]. These results suggest persons with MS do not participate in rates of physical activity necessary for health benefits, but there is no published data on the rates of physical activity across the lifespan, particularly among older adults with MS.

Of note, levels of physical activity are exceedingly low among the general population of older adults (≥ 60 years of age) [25,26]. For example, a recent study examined physical activity and sedentary behavior in older adults (55-86 years of age) and reported older adults engaged in approximately 13 minutes/day of MVPA compared with approximately 423 minutes/day of sedentary behavior [26]. To our knowledge, there is no research quantifying physical activity and sedentary behavior levels and rates in older adults with MS. Such information is necessary for understanding the degree of physical inactivity in this growing segment of the MS population and its associated implications for risks of comorbidity in older adults with MS.

The present study compared the continuum of objectively quantified levels of physical activity (i.e., sedentary behavior, light physical activity (LPA), and MVPA) and the rates of meeting public health guidelines for MVPA (i.e., ≥30 min/day) in young (i.e., ages 20-39 years), middle-aged (i.e., ages 40-59 years) and older adults (i.e., ages ≥60 years) in a large sample of persons with MS. We hypothesized decreased levels of physical activity, increased sedentary behavior, and lower rates of meeting guidelines for MVPA among older than younger adults with MS based on the pattern of physical activity seen for older adults from the general population.

## MATERIALS AND METHODS

### Participants

Over a 5-year period, persons with MS were recruited locally, regionally, and statewide throughout the Midwest and entire United States by sources including print and e-mail flyers and online advertisements on the National Multiple Sclerosis Society national and regional websites. We recruited participants for the study of physical activity behavior in MS. The inclusion criteria were: (1) definite diagnosis of MS; (2) relapse-free in the previous 30 days; (3) ambulatory with or without an assistive device; (4) 18 years of age or older; and (5) willingness to wear an accelerometer for 7 days. The final sample included 963 persons with MS, and all persons satisfied inclusion criteria and provided usable data for the analyses (≥2 days of valid accelerometer data) [27].

### Device

Participants wore an ActiGraph model 7164 accelerometer. The study, in sum, included nearly 100 accelerometers that were calibrated for accurately measuring physical activity prior to use by laboratory staff members walking on an treadmill (4.8kmph, 0% grade for 15 min) while wearing 4 to 8 accelerometers on a belt around the waist. Accuracy was confirmed by a <10% difference in average counts per minute across the 15 min period of walking among the batch of accelerometers worn simultaneously. The ActiGraph model 7164 accelerometer is a device that measures physical activity as activity counts per unit of time by using a piezoelectric bender element that produces an electrical signal proportionate to the force acting on it during movement. The electric signal is digitally converted into activity counts amalgamated over 1 min sampling intervals (i.e., 60 sec epochs); activity counts per minute are stored in the accelerometer’s random access memory. The data from the accelerometers were later downloaded and processed using ActiLife software. The data from each participant’s accelerometer were processed into 2 separate Microsoft Excel files representing wear time and time spent in sedentary, LPA, and MVPA based on activity count cut-points for persons with MS (i.e., sedentary: 0-99 counts/min; LPA: 100-1722 counts/min; MVPA: ≥1723 counts/min) [28]. Accelerometer wear time data were checked against participant recorded wear times from the log sheet, and only valid days (>10 h of wear time without periods of continuous zeros exceeding 60 min indicative of incompliance) were included in the analyses.

### Disability Status

The Patient-Determined Disease Steps (PDDS) scale was included as a self-report measure of MS disability status. The PDDS was developed as an inexpensive surrogate for the Expanded Disability Status Scale (EDSS) [29] and contains a single item ordinal scale for measuring self-reported neurological impairment, ranging from 0 (normal) through 8 (bedridden).

### Procedure

The procedures were approved by a university institutional review board and all participants provided written informed consent. After screening for inclusion criteria and provision of signed informed consent, all participants received an accelerometer, a log sheet, instructions for wearing the device, and the clinical, demographic, and PDDS scales. Participants were given written and graphic instructions to wear the accelerometer around the waist over the non-dominant hip during all waking hours of a 7-day period, except when swimming, bathing, or showering. Waking hours were defined as the moment of getting out of bed in the morning until the moment of getting into bed in the evening. During this 7- day period, participants were asked to maintain normal levels of physical activity. Participants were further asked to record the time of day the accelerometer was worn and any times throughout the day that the accelerometer was not worn. Participants returned study materials after the 7-day period.

### Data Analysis

All analyses were performed using SPSS version 21. Only participants with ≥2 valid days of accelerometer data were included in the analyses (n=963). We examined differences in demographic and clinical characteristics between young (i.e., ages 20-39), middle-aged (i.e., ages 40-59) and older adults (i.e., ages ≥60 years) using between-subjects analysis of variance (ANOVA) or chi-square (*χ^2^*) difference tests. The formation of these age groups is consistent with previous research in older adults (6). Descriptive statistics are presented in the text and tables as mean (SD) unless otherwise noted. The primary analysis involved a between-subjects ANOVA on accelerometer variables (i.e., wear time, minutes/day; number of valid days; sedentary behavior, min/day; LPA, min/day; MVPA; min/day; MVPA ≥30min/day, % of participants). This analysis further involved *post-hoc* decomposition with Bonferroni corrections for examining specific differences in accelerometer variables between age groups. We expressed group differences in mean scores based on Cohen’s *d* and interpreted the values as small, moderate, and large based on criteria of 0.2, 0.5, and 0.8, respectively [30]. Finally, we examined differences among age groups in percentage of persons with MS accruing ≥30 min/day of MVPA using a *χ^2^* test.

**Table 1:** Demographic and clinical characteristics in 963 persons with MS by years of age (i.e., 20-39, 40-59, and ≥60)

Variable	Age Groups			*p*-value
	Young (n=194)	Middle-aged (n=662)	Older (n=107)	
**Age, years**	33.1 (4.7)	49.6 (5.4)	63.0 (3.6)	0.001*
**Sex, % female**	82.2	85.5	77.6	0.09
**Race, % Caucasian**	91.2	94.2	96.3	0.20
**BMI, kg/m^2^**	27.6 (6.8)	27.1 (6.8)	27.6 (7.4)	0.24
**Education, % college graduate**	66.0	59.6	58.9	0.26
**Income, % >$40K/year**	60.1	73.3	63.6	0.001*
**Type of MS, %RRMS**	96.9	92.0	82.2	0.01*
**Disease duration, years**	6.3 (5.0)	10.8 (7.2)	16.5 (9.2)	0.001*
**PDDS score (mdn, IQR)**	1.0 (3.0)	2.0 (2.0)	3.0 (3.0)	0.001*

Note. Data presented as mean (SD), unless otherwise noted. MS=multiple sclerosis; RRMS=relapsing-remitting MS; PDDS=Patient Determined Disease Steps; BMI=body mass index;

* =Significance at *p*<0.05; Young adults=ages 20-39; Middle-aged=ages 40-59; Older adults=ages ≥60 years

## RESULTS

### Demographic and clinical characteristics

Demographic and clinical characteristics of the sample by age group (i.e., young, middle-aged, and older adults) are provided in Table 1. There were 194 young adults (i.e., ages 20-39 years), 662 middle-aged adults (i.e., ages 40-59 years), and 107 older adults (i.e., ages ≥60 years). There were significant differences (*p*<0.05) in age (years), income (% >$40K/year), type of MS (% RRMS), disease duration (years), and PDDS score among groups. There were no significant differences in sex (% female), race (% Caucasian), or BMI (kg/m^2^).

### Accelerometer wear time and number of valid days

Accelerometer wear time and number of valid days per age group (i.e., young, middle-aged, and older adults) are provided in Table 2. The ANOVA identified a statistically significant difference in number of valid days (*F*=11.1, *p*<0.05). The *post-hoc* analysis indicated that young and middle-aged adults with MS had fewer valid days than older adults with MS, and these were moderate-sized differences (*d*=0.60 and 0.43, respectively). There was no significant difference in number of valid days between young and middle-aged adults with MS. There were no significant differences in wear time (min/day) (*F*=2.9, *p*<0.05) among age groups.

### Sedentary behavior, LPA, and MVPA

**Figure 1.**
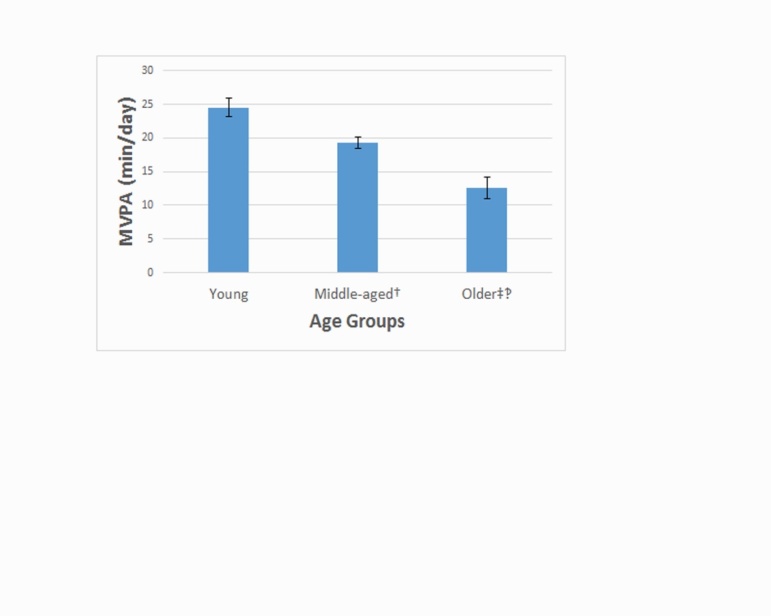
Minutes per day of moderate-to-vigorous physical activity (MVPA) among age groups of people with MS † = p < .05 for Young vs. Middle-aged groups; ‡ = p < .05 for Young vs. Older age groups; ‽ = p < .05 for Middle-aged vs. Older age groups; Young = age of 20-39 years; Middle-aged = age of 40-59 years; Older = ages ≥60 years. Values represent mean score and standard error of the mean.

Sedentary behavior, LPA, and MVPA per age group (i.e., young, middle-aged, and older adults) are provided in Table 2. The ANOVA identified a statistically significant difference in sedentary behavior (min/day) (*F*=8.0, *p*<0.05). The *post-hoc* analysis indicated that middle-aged and older adults with MS spent more time in sedentary behavior per day than young adults with MS, and these were small-to-moderate in magnitude (*d*=0.25 and 0.47, respectively). There was no difference in sedentary behavior between middle-aged and older adults with MS. The ANOVA identified a small, but statistically significant difference in MVPA (min/day) (*F*=12.6, *p*<0.05) and this is illustrated in Figure 1. The *post-hoc* analysis indicated that older adults with MS spent less time in MVPA per day than middle-aged and young adults with MS, and these were small-to-moderate in magnitude (*d*=0.36 and 0.64, respectively). Middle-aged adults with MS further spent less time in MVPA per day compared with young adults with MS and this difference was small in magnitude (*d*=0.26). There was no significant difference in LPA (min/day) among age groups (*F*=1.2, *p*>0.05).

**Table 2:** Accelerometer variables in 963 persons with MS by years of age (i.e., 20-39, 40-59, and ≥60)

Variable	Age Groups			*F*-value	*p*-value
	Young (n=194)	Middle-aged (n=662)	Older (n=107)		
**Wear time, min/day**	823.4 (70.8)	838.9 (86.1)	840.2 (70.8)	2.9	0.057
**Number of valid days**‡‽	5.8 (1.3)	6.0 (1.3)	6.5 (1.0)	11.1	0.001*
**Sedentary behavior, min/day**†‡	509.6 (83.7)	532.8 (100.4)	554.1 (89.9)	8.0	0.001*
**LPA, min/day**	289.4 (75.0)	287.7 (85.2)	275.0 (84.2)	1.2	0.302
**MVPA, min/day**†‡‽	24.5 (20.1)	19.3 (20.2)	12.6 (16.7)	12.6	0.001*

Note. Data presented as mean (SD), unless otherwise noted. LPA = light physical activity; MVPA = moderate-to-vigorous physical activity; Based on *post-hoc* Bonferroni corrections:

† *p* < .05 for Young vs. Middle-aged groups;

‡ *p* < .05 for Young vs. Older age groups;

‽ *p* < .05 for Middle-aged vs. Older age groups; Young adults=ages 20-39; Middle-aged=ages 40-59; Older adults=ages ≥60 years

### Physical activity guidelines for MVPA

There was a significant difference among age groups in percentage of persons with MS meeting public health guidelines for MVPA (i.e., accruing ≥30 min/day of MVPA) (*χ^2^*=9.1, *p*<0.05). Only 14.0% of older adults with MS accrue ≥30 min/day of MVPA compared with 20.8% and 28.4% of persons in middle-aged and young adults with MS, respectively.

## DISCUSSION

This study provided the first objective estimates of physical activity (i.e., LPA and MVPA) and sedentary behavior among older adults (i.e., ages ≥60) with MS. Our results demonstrate older adults with MS spend approximately 45 minutes per day in sedentary behavior more than young adults (i.e., ages 20-39) with MS. Our results further indicate that older adults with MS spend approximately 12 and 7 minutes per day in MVPA less than young and middle-aged adults (i.e., ages 40-59), respectively. Only 14% of older adults with MS meet public health guidelines for MVPA (i.e., ≥30 min/day of MVPA), and this was significantly lower when compared with approximately 21% and 28% of middle-aged and young adults with MS, respectively. However, there was no significant difference in minutes of LPA per day among age groups. Collectively, our data indicate older adults with MS engage in inadequate levels of physical activity and are more sedentary than middle-aged and young adults with MS. This is important for indicating that the growing population of older adults with MS could benefit from behavioral interventions for changing physical activity and sedentary behaviors with the objective of improving outcomes associated with MS and aging.

Older adults with MS (i.e., ages ≥60) seemingly engaged in similar levels of MVPA as older adults from the general population. For example, our results demonstrated that older adults with MS engaged in approximately 13 minutes of MVPA per day and this is comparable with a previous study that reported older adults from the general population engaged in approximately 14 minutes of MVPA per day [26]. However, older adults with MS are more sedentary than older adults in the general population. Older adults with MS spend approximately 554 minutes per day in sedentary behavior compared with approximately 423 minutes per day in older adults from the general population. Older adults with MS further engage in approximately 40 minutes less LPA per day compared with older adults in the general population [26]. Therefore, physical activity interventions that target a reduction of sedentary behavior and increases in LPA and MVPA may be particularly important for older adults with MS.

The growing cohort of older adults with MS undergoes normal age-related declines in physical and psychological functioning such as impairments in functional performance and increased disability [31]. However, these age-related declines are further compounded in older adults with MS by the progression of the disease, such that older adults with MS often report poor health status and reduced quality of life [32]. Although there is much research on the benefits of physical activity for young and middle-aged adults with MS, there is very little known about levels and rates of physical activity and the possible benefits in older adults with MS [19,21]. The results of this study, accompanied with the greater numbers of older adults living with MS than ever before, underscore the need for targeted physical activity interventions specific for this demographic of persons with MS [18].

The strengths of this study include the large sample size and the use of an objective measure for quantifying levels and rates of physical activity and sedentary behavior in older adults. However, there are some important limitations. We did not include a control, non-MS comparison group. We further did not analyze the physical activity and sedentary data of the older adults by demographic and clinical characteristics as the sample size did not permit such decompositions. Another limitation is the assumption that the activity count cut-points for sedentary, LPA, and MVPA are equally applicable across the age groups. If the assumption is incorrect (i.e., older adults expend more energy for MVPA compared with middle-aged and young adults), our estimates could biased. This could be a subject of future research. Further, future research could focus on identifying specific cut-points for LPA, as the large range of the category (i.e., 100-1722 counts/min) may be better distinguished by smaller subcategories. We believe additional research may be warranted that examines specific segments of the older adult MS population that may most benefit from physical activity interventions. The data from this study are also from a convenient sample that may not reflect the population characteristics of MS.

In conclusion, this study provides objective data on levels and rates of physical activity and sedentary behavior in older adults with MS. Overall, older adults with MS engage in less MVPA and more sedentary behavior compared with middle-aged and younger adults with MS. Older adults with MS further engage in less LPA and more sedentary behavior compared with data for older adults from the general population. We believe our results highlight the importance of developing physical activity interventions as an effective means for managing the progression of the disease in older adults with MS.
